# Picornavirus 2C proteins: structure-function relationships and interactions with host factors

**DOI:** 10.3389/fcimb.2024.1347615

**Published:** 2024-02-23

**Authors:** Chunhui Yin, Haomiao Zhao, Xiaoyi Xia, Zhengyang Pan, Daoqun Li, Leiliang Zhang

**Affiliations:** ^1^ Department of Clinical Laboratory Medicine, The First Affiliated Hospital of Shandong First Medical University & Shandong Provincial Qianfoshan Hospital, Jinan, Shandong, China; ^2^ Department of Pathogen Biology, School of Clinical and Basic Medical Sciences, Shandong First Medical University & Shandong Academy of Medical Sciences, Jinan, Shandong, China; ^3^ Medical Science and Technology Innovation Center, Shandong First Medical University & Shandong Academy of Medical Sciences, Jinan, Shandong, China

**Keywords:** picornavirus, 2C protein, innate immunity, antiviral drug, viral replication

## Abstract

Picornaviruses, which are positive-stranded, non-enveloped RNA viruses, are known to infect people and animals with a broad spectrum of diseases. Among the nonstructural proteins in picornaviruses, 2C proteins are highly conserved and exhibit multiple structural domains, including amphipathic α-helices, an ATPase structural domain, and a zinc finger structural domain. This review offers a comprehensive overview of the functional structures of picornaviruses’ 2C protein. We summarize the mechanisms by which the 2C protein enhances viral replication. 2C protein interacts with various host factors to form the replication complex, ultimately promoting viral replication. We review the mechanisms through which picornaviruses’ 2C proteins interact with the NF-κB, RIG-I, MDA5, NOD2, and IFN pathways, contributing to the evasion of the antiviral innate immune response. Additionally, we provide an overview of broad-spectrum antiviral drugs for treating various enterovirus infections, such as guanidine hydrochloride, fluoxetine, and dibucaine derivatives. These drugs may exert their inhibitory effects on viral infections by targeting interactions with 2C proteins. The review underscores the need for further research to elucidate the precise mechanisms of action of 2C proteins and to identify additional host factors for potential therapeutic intervention. Overall, this review contributes to a deeper understanding of picornaviruses and offers insights into the antiviral strategies against these significant viral pathogens.

## 1 Introduction

Picornaviruses are positive-stranded, non-enveloped RNA viruses and are responsible for a variety of human and animal diseases. Members of picornavirus include enterovirus 71 (EV-A71), poliovirus (PV), coxsackievirus (CV), rhinovirus (RV), and hepatitis A virus (HAV) (Zell et al., 2017). These viruses cause high morbidity in humans, infecting the central nervous system, heart, skin, eyes, and other internal organs (Stanway et al., 2000; Grubman and Baxt, 2004; Tapparel et al., 2013; Vaughan et al., 2014). Hundreds of millions of people suffer from picornavirus infections each year, placing a significant financial burden on healthcare systems. (GBD 2017 Disease and Injury Incidence and Prevalence Collaborators, 2017) Picornaviruses also encompass the foot and mouth disease virus (FMDV) and encephalomyocarditis virus (EMCV). FMDV primarily affects livestock and has minimal impact on human health, while EMCV can infect humans but with low rates of morbidity (Zell et al., 2017).

Like most viruses, picornaviruses follow a typical life cycle consisting of attachment, entry, uncoating, replication, translation, assembly, and release. Initially, picornaviruses attach to specific receptors on the surface of host cells, which facilitates virus enrichment and enhances infectivity (Heckenberg et al., 2022). Subsequently, the virus is internalized into endosomes, where it undergoes decapsidation and releases RNA into the cytoplasm (Baggen et al., 2018). This RNA then serves as a template for the translation of viral polyproteins and replication of the viral genome.

**Figure 1:**
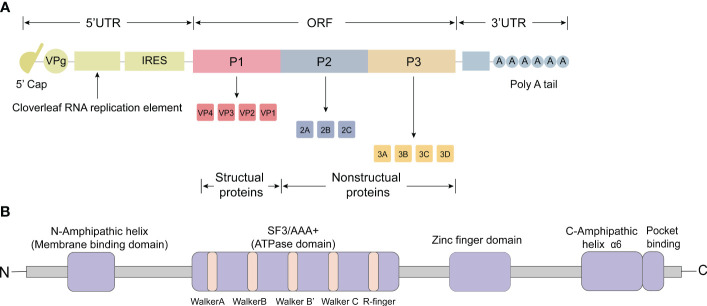
Genomic organization and functional motifs of the picornavirus 2C protein. **(A)** The picornavirus genome consists of an open reading frame (ORF) and two untranslated regions (UTRs) located at the 5’ and 3’ ends. The 5’ UTR contains the cloverleaf replication element and the internal ribosome entry site (IRES). The ORF encodes a single, multimeric protein and can be divided into three distinct regions: P1, P2, and P3. Processing of P1 leads to the production of structural proteins that collectively form the viral capsid (VP1, VP2, VP3 and VP4). Processing of P2 results in the formation of non-structural proteins 2A, 2B and 2C, while processing of P3 leads to the production of non-structural proteins 3A, 3B, 3C, and 3D. **(B)** The 2C protein comprises various functional motifs, including amphipathic α-helices (AH domain) at the N-terminus, conserved superfamily 3 (SF3) motifs A, B, C and ‘R finger’, a zinc finger domain, C-terminus amphipathic domain (α6), and a pocket binding domain (PBD).

The picornavirus genome has a positive-strand polarity and ranges in length from 6.7 to 10.1 kilobases (Baggen et al., 2018). The first simple model of PV was produced in 1959 based on X-ray diffractograms, and a near-atomic 3D structure of PV was later determined in 1985 with a resolution of 2.9 Å (Finch and Klug, 1959; Hogle et al., 1985). The 5’ untranslated region (UTR) includes the cloverleaf replication element, the internal ribosome entry site (IRES), which is followed by a significant open reading frame (ORF) responsible for encoding a single, multimeric protein (**Figure 1A**). This protein is co-translated and undergoes post-translational processing, both *in cis* and *trans*, facilitated by the viral protease 2Apro and 3CDpro (Martínez-Salas and Ryan, 2010). Within the ORF, there are three distinct regions: P1, P2, and P3 (**Figure 1A**). The processing of P1 results in the production of structural proteins, which collectively form the viral capsid (VP1, VP2, VP3, and VP4) (**Figure 1A**) (Kitamura et al., 1980). Notably, the capsid proteins VP1 to VP4 were listed in order of their apparent molecular weights in SDS polyacrylamide gel (SDS-PAGE) electrophoresis from largest to smallest. The processing of P2 leads to the formation of non-structural proteins 2A, 2B, and 2C, while the processing of P3 results in the production of non-structural proteins 3A, 3B, 3C, and 3D (**Figure 1A**). Moreover, these processes generate functional intermediates such as 2BC, 3AB, and 3CD (Kräusslich et al., 1988). Additionally, a poly(A) tail is situated at the 3’ end of the RNA molecule (Yogo and Wimmer, 1972; Dorsch-Häsler et al., 1975).

Picornavirus replication takes place on intracellular membranes that have been modified by the virus. These membranes serve as physical support and provide a favorable lipid environment for viral RNA assembly and replication (Miller and Krijnse-Locker, 2008; Altan-Bonnet, 2017). The replication process involves two steps: the synthesis of negative-strand RNA and positive-strand RNA, respectively. In the first step, the RNA-dependent RNA polymerase 3D^pol^ transcribes the positive-strand RNA, which is released from the viral capsid, into complementary negative-strand RNA (Kempf and Barton, 2015). This synthesis is initiated by VPg and its uridylated derivatives, using the 3-terminal poly(A) sequence in viral RNA templates. The result is the production of a poly(U) product linked to VPg at the 5-terminal end of the negative-strand RNA intermediates (Paul and Wimmer, 2015). The next step involves generating new positive-stranded RNA from the produced negative-stranded RNA. This process is initiated by Uridylylated VPg (VPgpUpUOH) at the complementary adenosine base located at the 3-terminal end of the negative-stranded RNA template (Sun et al., 2014). The resulting new positive-stranded RNA can be utilized as a template for further translation and RNA replication. Alternatively, it can be glycosylated to form new infectious viral particles, which are assembled with viral capsid proteins and nonstructural proteins. These viral particles then exit the host cell through the cell lysis pathway to initiate a new life cycle.

## 2 Domains of 2C proteins

### 2.1 The amphipathic α-helices

After invading host cells, positive-stranded RNA viruses often modify the host cell membrane to facilitate their attachment to the host cell membrane and the synthesis of daughter RNAs. One important nonstructural protein, 2C, found in picornaviruses, contains amphipathic α-helices (AH domain) at its N-terminus, which help target the host cell membrane (**Figure 1B**) (Paul et al., 1994; Teterina et al., 2006).

Initially, it was discovered that the expression of PV 2C or 2BC induced endoplasmic reticulum (ER) membrane rearrangement, but it was not dependent on the ATPase activity (Cho et al., 1994). Subsequently, it was found that the N-terminal fragment of PV 2C (amino acids 1-77) promoted the targeting of soluble chloramphenicol acetyltransferase (CAT) to the ER membrane (Echeverri et al., 1998), and the expression of a fragment containing the initial 122 amino acids of PV 2C induced membrane reorganization (Teterina et al., 1997). Additionally, the PV 2C protein was found to possess an N-terminal amphipathic α-helix (amino acids 19-36) (Paul et al., 1994; Teterina et al., 2006). This N-terminal amphipathic domain is conserved among picornaviruses, as similar helical structures are found in the 2C proteins of EV-A71, CV-A18, and human rhinovirus (HRV) (Teterina et al., 2006).

The amphipathic domain of the 2C protein serves as a tethering point for attaching viral replication proteins to the membrane. Investigations have demonstrated that PV 2C proteins specifically target to host lipid droplets (LDs). PV 2C proteins lacking the N-terminal 1-39 amino acids (2C 40-329) or only the amphipathic α-helix (2C Δ17-38) are unable to bind to LDs. Conversely, the presence of PV 2C 1-38 ensures the binding capacity of 2C to LDs, underscoring the pivotal role of the amphipathic domain in directing 2C to this location (Laufman et al., 2019).

In addition to assisting PV 2C in binding to liposomes and targeting LDs membranes, the amphipathic domain could also remodel spherical liposomes into tubular liposomes (Echeverri et al., 1998; Varkey et al., 2020). This remodeling activity may be involved in endosomal membrane remodeling within the host cell. The membrane remodeling ability of the PV 2C amphipathic domain shares similarities with other proteins such as α-synuclein, trypsin, pancreatic amyloid polypeptide, and BAR structural domain proteins (Zimmerberg and Kozlov, 2006; Frost et al., 2008; Varkey et al., 2010).

It has been observed that mutations in the hydrophobic surface of the amphipathic domain significantly impair its membrane remodeling ability. These mutations also lead to a substantial reduction in viral plaque formation during PV replication. These findings highlight the significance of membrane remodeling by amphipathic α-helices, which may be critical for the formation of viral replication complexes (RCs) (Varkey et al., 2020).

### 2.2 ATPase domain

According to predictions, PV 2C is classified as an SF3 helicase (Pfister and Wimmer, 1999; Varkey et al., 2020). 2C from PV and EV-A71 possess a middle domain of 137 amino acids, which includes the conserved superfamily 3 (SF3) signature A, B and C motifs (**Figure 1B**) (Wang et al., 2014; Xia et al., 2015). These motifs provide the basis for its RNA helicase activity along with the R finger (R240 and R241) (Guan et al., 2017). Motifs A and B are known as ‘Walker’ NTP binding motifs, commonly found in proteins that bind ATP and/or GTP (Rodríguez and Carrasco, 1993). Motif C is considered as a characteristic feature of SF3 helicases (Pfister and Wimmer, 1999).

Guan et al. reported that the C-terminal helix of the PV 2C protein could mediate self-oligomerization similar to that induced by EV-A71 2C through a 2C-2C-specific interaction. Additionally, this self-oligomerization of 2C is essential for its ATPase activity (Guan et al., 2018). Mutations at various sites, such as L327 and F328 in the pocket-binding domain (PBD), K135A and D176N in the Walker A and B motifs, and R240 and R241 to either A or K (Guan et al., 2017, 2018), have been found to eliminate the ATPase activity and homo-oligomerization capacity of PV 2C and EV-A71 2C. As a result, replication and infection of viruses are inhibited. These mutations highlight the importance of PBD, R finger, and Walker motifs for the ATPase activity of 2C.

It was found that structurally, the 2C proteins of PV and EV-A71 have similar conformations and catalytic residues within the ATPase active site, and thus they are presumed to resemble each other functionally (Mirzayan and Wimmer, 1994; Pfister and Wimmer, 1999). Additionally, the ATPase and deconjugase activities were inhibited in the EV-A71 2C GK134AA, leading to termination of RNA replication and virus production. These RNA remodeling activities were also present in the PV 2C and CV-A16 2C ATPases. This suggests that RNA remodeling activities facilitated by 2C ATPase are critical in the RNA replication process and viral life cycle of Picornaviruse. Further studies have demonstrated that the C-terminal is essential for the deconjugating enzyme activity and that the structural domain bound to RNA is necessary for the RNA chaperoning function of the 2C ATPase (Xia et al., 2015).

RNA remodeling proteins can be categorized into three groups: ATP-dependent RNA helicases, ATP-independent RNA haptens, and RNA annealing agents. These proteins can remodel RNA structure, perhaps altering RNA-protein interactions. Based on conserved motifs, ATP-dependent RNA helicases are categorized into six superfamilies (SF1 to SF6) (Kadaré and Haenni, 1997). They can use the energy generated by ATP binding and/or hydrolysis to move along the RNA double strand, contributing to ATP-dependent structural RNA remodeling (Xia et al., 2015). Conversely, RNA haptens that do not require ATP destabilize RNA by reducing the thermodynamic stability of the RNA double strand, which promotes the formation of the appropriate RNA structure. Some evidence suggests that they achieve this by altering the dynamics of the RNA structure and rapidly separating from the RNA (Lorsch, 2002; Rajkowitsch et al., 2007; Grohman et al., 2013).

The discovery made by Xia et al. in 2015 revealed that the ATPase domain of EV-A71 2C possesses RNA remodeling activity, enabling it to act as both an ATP-dependent RNA helicase and an ATP-independent RNA chaperone. This activity promotes viral RNA synthesis from the viral RNA template *in vitro*, which is mediated by EV-A71 RdRP. Remarkably, this function is also exhibited by the 2C proteins of other viruses like CV-A16 (Xia et al., 2015) It is noteworthy that only eukaryotically expressed 2CATPase proteins displayed RNA double-stranded body unwinding activity, and their expression level was significantly higher than that of prokaryotic expression.

Using PV 2C and its derivatives, researchers have performed the first biochemical characterization of the determinants that drive the specificity and catalytic efficiency of picornavirus 2C ATPase (Yeager et al., 2022). Their findings revealed that RNA and DNA, propelled by the phosphodiester backbone, attach to the same or overlapping sites on 2C. However, only RNA triggers ATP hydrolysis. Therefore, it is hypothesized that upon binding of backbone-driven RNA to 2C, reorientation of the ribose hydroxyl group occurs, resulting in catalytic capacity. 2C also binds to ATP by a two-step mechanism (Yeager et al., 2022). The first step is catalyzed by α and β phosphates of ATP, and the second one is activated by the adenine and other substituents of ATP.

The IRES in picornaviruses is highly structured and integral to the RNA genome (Cheng et al., 2013; Yeager et al., 2022). Firstly, IRES may function during viral replication through the RNA chaperone activity of 2C ATPases. This interaction facilitates the correct folding and refolding of the RNA strand following annealing, thereby finalizing the replication process of the resulting RNA strand Secondly, the RNA chaperone activity of 2C assists in unraveling intermediate dsRNA, facilitating the recycling efficiency of viral RNA templates. For instance, EV-A71 2C precisely facilitates 3D-mediated RNA synthesis *in vitro* through the mentioned mechanism (Xia et al., 2015). Impairment of RNA replication and diminished viral viability are commonly observed when the ATPase and depolymerase activities in EV-A71 2C are defective.

The AAA+ ATPase superfamily performs its functions through a hexameric ring structure (Enemark and Joshua-Tor, 2006; Cheng et al., 2013). 2C proteins belong to the SF3 superfamily and also form a hexameric ring. Thus, further studies on structural domains like the hexameric ring, PBD, R finger, and Walker motifs will contribute to understanding the ATPase and deconjugating enzyme activities of 2C and pave the way for studying antiviral drug targets.

### 2.3 Zinc finger domain

Initially, scientists discovered that the C-terminal end of the 2C protein is rich in cysteine motifs and could bind to zinc *in vitro* (**Figure 1B**) (Teterina et al., 1997; Pfister et al., 2000). Further studies found most of the other Picornavirus 2C proteins have cysteine-rich sites (CRS) in the corresponding regions, such as EV-A71, PV, RV, and CV-B (Guan et al., 2018), except in HAV and FMDV where there was no evidence supporting the presence of CRS (Pfister et al., 2000; Sweeney et al., 2010).

The zinc ion in PV 2C proteins is coordinated by C269, C272, C281, and C286 (Guan et al., 2018). This coordination exhibits uncommon triangular biconical geometry. The zinc finger folding of 2C is distinctive, as crystallographic studies have shown that the zinc finger structures of PV 2C and EV-A71 2C do not belong to any of the eight known zinc finger folding groups (Guan et al., 2017, 2018). Therefore, they are classified in the ‘EV 2C-like’ folding group.

Unlike other structural domains, the zinc finger domain in 2C proteins is not conserved. Research suggests four potential zinc coordination sites (PCS) in the cysteine-rich motif of PV 2C, which are typical CCCC-type zinc fingers (Pfister et al., 2000). The cysteine residues in these PCSs are preserved as zinc-liganding residues. While EV-A71 2C comprises of PCS1, PCS3, and PCS4, PCS2 is missing (Guan et al., 2017). Interestingly, crystallographic data reveals that HRV 2C also lacks PCS2, but it does not appear to affect its zinc-liganding ability (Chen et al., 2022). Many other enteroviruses also lack cysteine or histidine in PCS2. However, the absence of PCS2 does not diminish its importance, as residues N273 and N274 in PCS2 of PV 2C, despite not directly contacting zinc, are crucial for PV replication (Pfister et al., 2000).

Studies suggest that zinc fingers can play a role in stabilizing protein folding, mediating binding interactions, and conferring specificity in protein-protein interactions. Additionally, the four PCSs have distinct functions. The maintenance of the zinc finger and hexamer folding is a crucial function of PCS1 and PCS3. Conversely, PCS2 and PCS4 may have an involvement in interacting with other proteins during encapsulation (Guan et al., 2018). Moreover, PV 2C’s PCS2 has been linked to a temperature-sensitive phenotype and a defect in encapsulation. However, attempts to increase the infectivity of EV-A71 2C zinc fingers by introducing PCS2 through mutation were unsuccessful (Klein et al., 2000; Wang et al., 2014).

Multiple residues and sites can influence 2C function by impacting the zinc finger domain. For instance, the PV 2C protein’s 269-286 region is vital for zinc binding (Klein et al., 2000). The N- and C-terminal amphipathic helices of 2C anchor the protein to the membrane and participate in zinc binding (Paul et al., 1994; Teterina et al., 1997; Wang et al., 2014). C270, C281, and C286 are crucial for correctly folding the EV-A71 2C protein (Guan et al., 2017). Wang et al. found that mutating a cysteine or histidine to alanine in the zinc-binding structural domain of PV 2CATPase, specifically A138V, M293V, and K295R, resulted in severe RNA replication defects. At 37°C, the temperature-sensitive mutant (C272A/H273A) showed evidence of clathration and a potential defect in encapsulation. This indicates that the C-terminal cysteine-rich region of 2CATPase plays a role in clathration, potentially through interaction with the upstream segment located between the nucleotide-binding structural domains of boxes A and B (Wang et al., 2014).

## 3 Structures of enterovirus 2C

### 3.1 EV-A71

**Figure 2:**
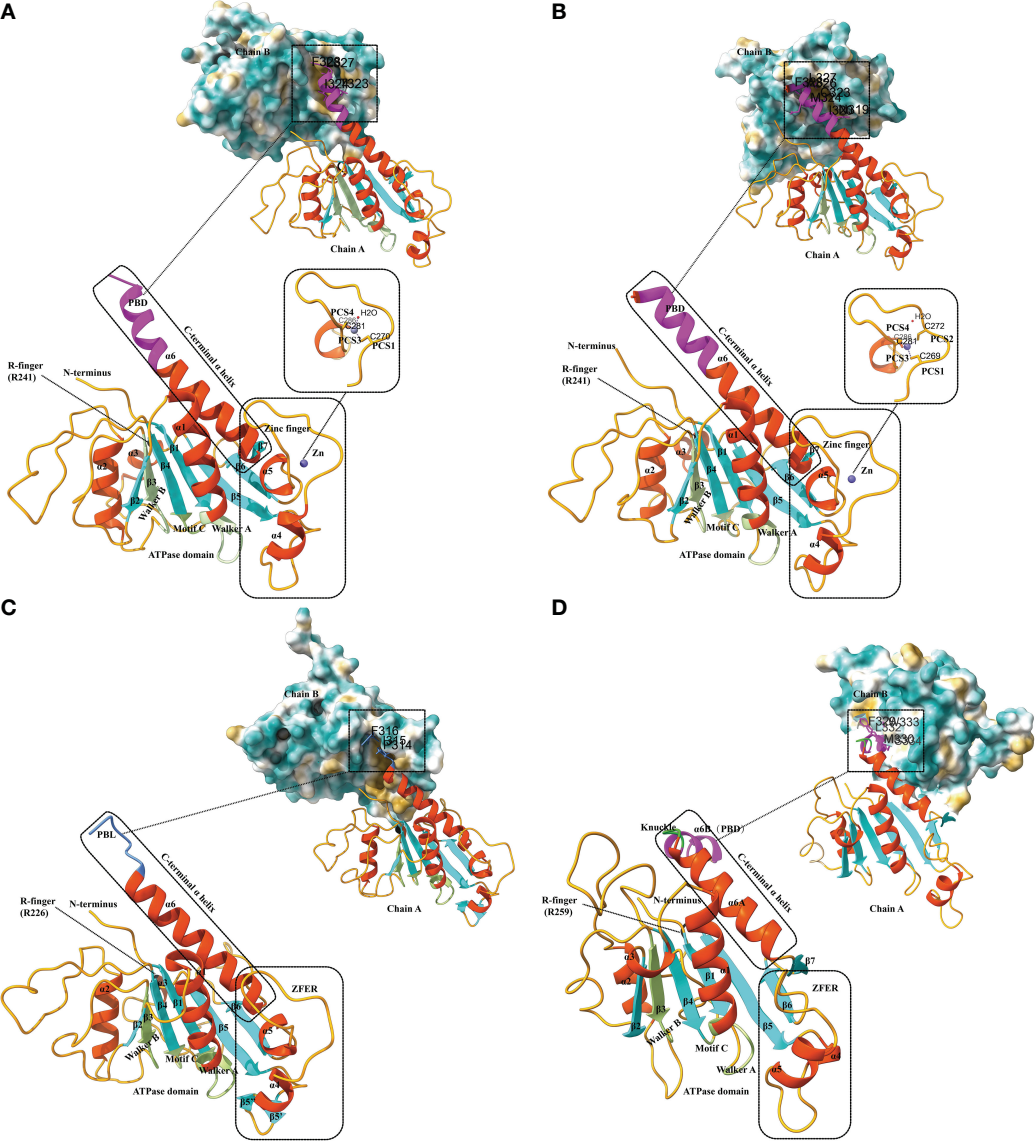
Structural characteristics of different 2C proteins from EV-A71, PV, FMDV and HAV. **(A)** Ribbon model of EV-A71 2C (PDB ID: 5GRB). The secondary structure elements are labeled and colored as follows: α helices in orange-red, β sheets in cyan, and loops in orange. The conserved ATPase motifs Walker A and Walker B, motif C, and arginine are highlighted in fluorescent green and labeled with A, B, C, and R-finger, respectively. The zinc-binding site of EV-A71 2C is shown with a stick model, where residues involved in zinc coordination are depicted. The pocket-binding domain (PBD) is represented in magenta. Views at the 2C-2C juncture illustrate the interaction between the PBD and the hydrophobic pocket observed in the structures of EV-A71 2C. The three-dimensional structures depicted in the figures were generated using Discovery Studio 2016 Client and ChimeraX. **(B)** Ribbon model of PV 2C (PDB ID: 5Z3Q). The secondary structure elements are labeled and colored as follows: α helices in orange-red, β sheets in cyan, and loops in orange. The conserved ATPase motifs Walker A and Walker B, motif C, and arginine are highlighted in fluorescent green and labeled with A, B, C, and R-finger, respectively. The zinc-binding site of PV 2C is shown with a stick model, where residues involved in zinc coordination are depicted. The PBD is represented in magenta. Views at the 2C-2C juncture illustrate the interaction between the PBD and the hydrophobic pocket observed in the structures of PV 2C. **(C)** Ribbon model of FMDV 2C (PDB ID: 7E6V). The secondary structure elements are labeled and colored as follows: α helices in orange-red, β sheets in cyan, and loops in orange. The conserved ATPase motifs Walker A and Walker B, motif C, and arginine are highlighted in fluorescent green and labeled with A, B, C, and R-finger, respectively. The zinc finger equivalent region (ZFER) and the pocket-binding loop (PBL) are depicted in cornflower blue. Views at the 2C-2C juncture illustrate the interaction between the PBL and the hydrophobic pocket observed in the structures of FMDV 2C. **(D)** Ribbon model of HAV 2C (PDB ID: 7XT3). The secondary structure elements are labeled and colored as follows: α helices in orange-red, β sheets in cyan, and loops in orange. The conserved ATPase motifs Walker A and Walker B, motif C, and arginine are highlighted in fluorescent green and labeled with A, B, C, and R-finger, respectively. The ZFER and the knuckle-shaped helices at the C terminus of HAV 2C are shown in lime color, where the two helices, α6A and α6B, are oriented nearly perpendicularly to each other, forming a “knuckle.” The PBD is represented in magenta. Views at the 2C-2C juncture illustrate the interaction between the PBD and the hydrophobic pocket observed in the structures of HAV 2C.

EV-A71 is the major pathogen responsible for hand-foot-mouth disease. The EV-A71 2C structure, reported by Guan et al. in 2017, represents the first high-resolution Picornavirus 2C structure to be documented (**Figure 2A**) (Guan et al., 2017). The EV-A71 2C is composed of three domains: an ATPase domain, a zinc finger, and a C-terminal α helix. The ATPase structural domain presents a typical α/β Rossmann fold, sequentially consisting of helix α1, a five-stranded parallel β-sheet (β1 to β5), and helices α2 and α3 (Guan et al., 2017). Walker A and B are located within the ATPase domain; motif A is found between β1 and α1, whereas motif B is found between β3 and α2 (**Figure 2A**). The motif C is located between β4 and α3. The zinc finger, a cysteine-rich motif, is located at 270 to 286. It is linked to the ATPase structural domain through an antiparallel β-sheet interaction between β5 and β6. Exceptionally, the zinc coordination of EV-A71 2C possesses a tridentate bipyramidal geometry, which is a rare feature in zinc fingers (Krishna et al., 2003; Laitaoja et al., 2013). In EV-A71 2C, the three Sγ atoms of C270, C281, and C286 form an equilateral triangle and lie in the same plane as the zinc. And its PCS critically differs from picornavirus 2C homologs, with only PCS1, PCS3 and PCS4, and no PCS2. Finally, the C-terminal helix α6 of EV-A71 2C binds to the hydrophobic pockets of neighboring 2C molecules in a ‘knob-into-hole’ manner, mediating 2C-2C oligomerization and facilitating the formation of membrane-bound RNA RCs (**Figure 2A**) (Guan et al., 2017). The T323, I324, L327, and F328 side chains in helix α6 constitute the PBD, which is located between the zinc finger and the ATPase helicase domain and consists mainly of hydrophobic residues. The salt bridge between E325 and R144 of the PBD enhances interactions between neighboring 2C molecules (Guan et al., 2017).

### 3.2 PV

PV is the pathogen of poliomyelitis, and its infection can directly damage motor neurons, leading to fever, limb pain, and flaccid paralysis (Couderc et al., 1989). Paralytic poliomyelitis is a disorder typically caused by poliovirus, which manifests as irregular and flaccid paralysis of varying severity, often occurring in young children. Understanding the structure and viral life cycle remains critical for the development of antiviral drugs, and the 2C protein is an important target for the development of antiviral agents (DAAs) (Bauer et al., 2017).

There is a high level of similarity between PV 2C and EV-A71 2C. Both of them have 329 residues and are similar in overall folding (Guan et al., 2018). PV 2C can be categorized into an N-terminal domain, a central ATPase domain, a zinc finger domain, and a long helical C-terminal domain (**Figure 2B**). The PV 2C N-terminal structural domain is highly composed and contains a membrane-binding motif (Echeverri et al., 1998), an amphipathic helix (Paul et al., 1994), an oligomerization motif (Adams et al., 2009), and an RNA-binding motif (Rodríguez and Carrasco, 1995). The amino acid sequences of PV 2C ATPase and EV71 2C deconjugating enzyme are relatively similar, but evidence suggesting that PV 2C has functional deconjugating enzyme activity remains in short. In structure, their ATPase domains both adopt the typical α/β Rossmann fold. In composition, they similarly contain three α-helices (α1-α3) and a five-stranded parallel β-sheet (β1-β5). The cysteine-rich motif (residues 267-289) of PV 2C is located between α4 and β6 and folds into a zinc finger domain, which serves as an intermediate region connecting the ATPase domain to the C-terminal domain of the long helix (**Figure 2B**). However, unlike PV 2C, which is a CCCC-type zinc finger (C269, C272, C281, and C286), many EV 2Cs, including EV-A71 2C, do not have PCS2 (Guan et al., 2018). In addition, there is a hydrophobic interaction between zinc finger and the ATPase domain that stabilizes the folding of zinc finger (**Figure 2B**). The C-terminal of PV 2C is an amphipathic long helix. This region accomplishes 2C-2C self-oligomerization (most likely hexamerization) by occupying hydrophobic pockets located on adjacent PV 2C ATPases in the lattice, in other words, through PBD interactions, similar to the EV-A71 2C-2C interactions that we mentioned before (**Figure 2B**). This is the basis for the ATPase activity of 2C, and F238A and L327A play a key role in the process. Additionally, the C-terminal α6 helix of the PV 2C also lacks the same hinge area as the EV-A71 2C, which means that it must remain straight (Guan et al., 2018).

### 3.3 FMDV

FMDV is the causative agent of foot and mouth disease, a highly contagious viral infection that affects cloven-hoofed animals. Due to the highly contagious character of FMDV, concerns have been raised about its outbreaks and its potential public health risks (Jamal and Belsham, 2013). 2C proteins are non-structural proteins that play an important role for FMDV, and researchers are developing a deeper understanding of FMDV through the use of 2C proteins (Grubman and Baxt, 2004).

The crystal structure of the FMDV 2C fragment has a resolution of 1.83 Å and consists of an ATPase domain, a ZFER-equivalent region, and a C-terminal domain (**Figure 2C**) (Zhang et al., 2022). Similar to EV-A71 2C, the ATPase domain presents a typical α/β Rossmann fold, sequentially consisting of helix α1, a five-stranded parallel β-sheet (β1 to β5), and helices α2 and α3s. But the electron density map of the FMDV 2C ATPase shows that it is partially missing in the loop between β3-α2, β4-β5, and β6-α6 (**Figure 2C**). Between β5 and β6 of FMDV 2C, a long loop structure similar to the zinc finger of EV 2C exists. Because it lacks Cys or His required for zinc coordination, it can only be referred to as a zinc finger equivalent region (ZFER). Unlike other picornaviruses, the PBD in the C-terminal helical domain of FMDV 2C evolves into a loop (PBL) (**Figure 2C**). It can also occupy the hydrophobic pocket of neighboring 2C and mediate the oligomerization of FMDV 2C (Zhang et al., 2022). Conclusively, the difference of 2C between FMDV and other picornaviruses lies in the ZFER of FMDV 2C lack of zinc-ligand residues, and PBL in the C-terminal rather than a PBD.

### 3.4 HAV

HAV is the only Hepatovirus member of the *Picornaviridae* family of viruses. It is common for children and adolescents to be infected with HAV, and symptoms are usually mild, consisting mainly of malaise and loss of appetite. Nonetheless, a small percentage of middle-aged and older (over 50 years of age) HAV-infected patients may die from fulminant hepatitis.

The crystal structure of HAV 2C (resolution 2.2 Å) also contains an ATPase structural domain, a ZFER domain, and a PBD domain in C-terminal (**Figure 2D**). However, as a specific picornavirus, its 2C protein differs from those of other picornaviruses. The ATPase domain of HAV 2C consists of a five-stranded parallel β-plane (β1-β5), and the loops connecting β3-β2, β2-β4 and β4-β5 are partially disordered (**Figure 2D**) (Chen et al., 2022). Although HAV 2C exhibits ATPase and RNA-binding activities, it has not been reported to have double-stranded deconjugation activity (Xia et al., 2015).

The ZFER region of HAV 2C does not contain Cys-rich motifs necessary for forming a zinc finger structure. In contrast to the 41-residue zinc finger found in PV 2C and EV-A71 2C, the ZFER region of HAV 2C is smaller and more compact, consisting of only 30 residues (Chen et al., 2022). And it may not require zinc coordination to maintain structural stability. The C-terminal helix of HAV 2C is two nearly perpendicular knuckle-shaped helices, distinct from the single long helix at the C-terminal end of EV-A71 2C and PV 2C (**Figure 2D**). HAV 2C-2C interaction involves a larger molecular surface, including two contact sites and a hydrophobic pocket formed between the ATPase domain and the ZFER of the neighboring 2C. Moreover, although the orientation of HAV and 2C of EV during self-polymerization is different, the pattern of PBD-Pocket interaction remains similar. Additionally, it was found that the second contact site, the α6B helix, is essential for 2C self-polymerization (Chen et al., 2022).

## 4 Functions of 2C

### 4.1 The role for 2C in viral morphogenesis

2C performs a key function during viral morphogenesis, especially in the interaction with capsid proteins (VP1 and VP3) (Wang et al., 2012a). A studies by Wang et al. confirmed that the cysteine-rich structural domain of PV 2C near the C-terminus also functions in capsidization (Wang et al., 2014). Various sites within the 2C protein are essential for capsidization. For instance, using the PV/CV-A20 chimera, Liu et al. that N252 of PV 2C can interact with E180 of the CV-A20 protein and thus participate in capsidization (Liu et al., 2010). Additionally, the PV 2C mutant K259A has been found to play a crucial role in the subsequent cycle of capsidization and the uncoating process (Asare et al., 2016). The Q65, L125, and V218 mutations in PV 2C also affect capsidization (Vance et al., 1997; Wang et al., 2014; Asare et al., 2016).

PV 2C exhibits temperature-sensitive properties, which can be attributed to specific amino acid residues, namely V218, M246 and I248 (Li and Baltimore, 1988; Dove and Racaniello, 1997). Interestingly, the temperature-sensitive phenotype of the PV 2C is closely linked to the process of viral capsidization and uncoating. Two revertant mutants, 2C-31 R1 and 2C-31 R3, have been constructed, and it was discovered that 2C-31 R1 shows a defect in uncoating at 32°C (Li and Baltimore, 1990). And after a short incubation at 39°C and growth at low temperatures, this defect is overcome. Further research reveals that nucleotides 5000 and 5007 sites are located downstream of the insertion junction in the coding sequence of PV 2C, and their mutation leads to changes in conserved amino acid residues of PV 2C polypeptide, ultimately causing uncoating defects. The other mutant, PV 2C-31 R3, appears to contain a secondary revertant mutant of an uncoating-deficient intragenic repressor and does not exhibit an uncoating defect. Importantly, the combination of the virus with the cellular receptor is not affected by the uncoating defect (Li and Baltimore, 1990). Furthermore, temperature-sensitive clathrate defects in the PV 2C ATPase K259A are linked to delayed deconjugation of viral particles at 33°C during the subsequent transfection cycle (Asare et al., 2016).

2C proteins can also interact with host proteins to promote viral morphogenesis. Studies have shown that Heat shock protein 8 (HSPA8) and HSPA9 can interact with EV-A71 2C to facilitate viral particle assembly (Su et al., 2020). The temperature-sensitive phenotype of the 2C protein affects RNA replication, resulting in delayed virus growth, protein synthesis, and the production of mature viruses. Viral inhibitors targeting virus encapsidation, such as hydantoin, have been developed. Besides inhibiting viral capsidization (Vance et al., 1997), hydantoin also prevents PV viral protein cleavage after protein synthesis (Verlinden et al., 2000).

### 4.2 2 Host cell proteins interact with 2C for efficient replication

**Figure 3:**
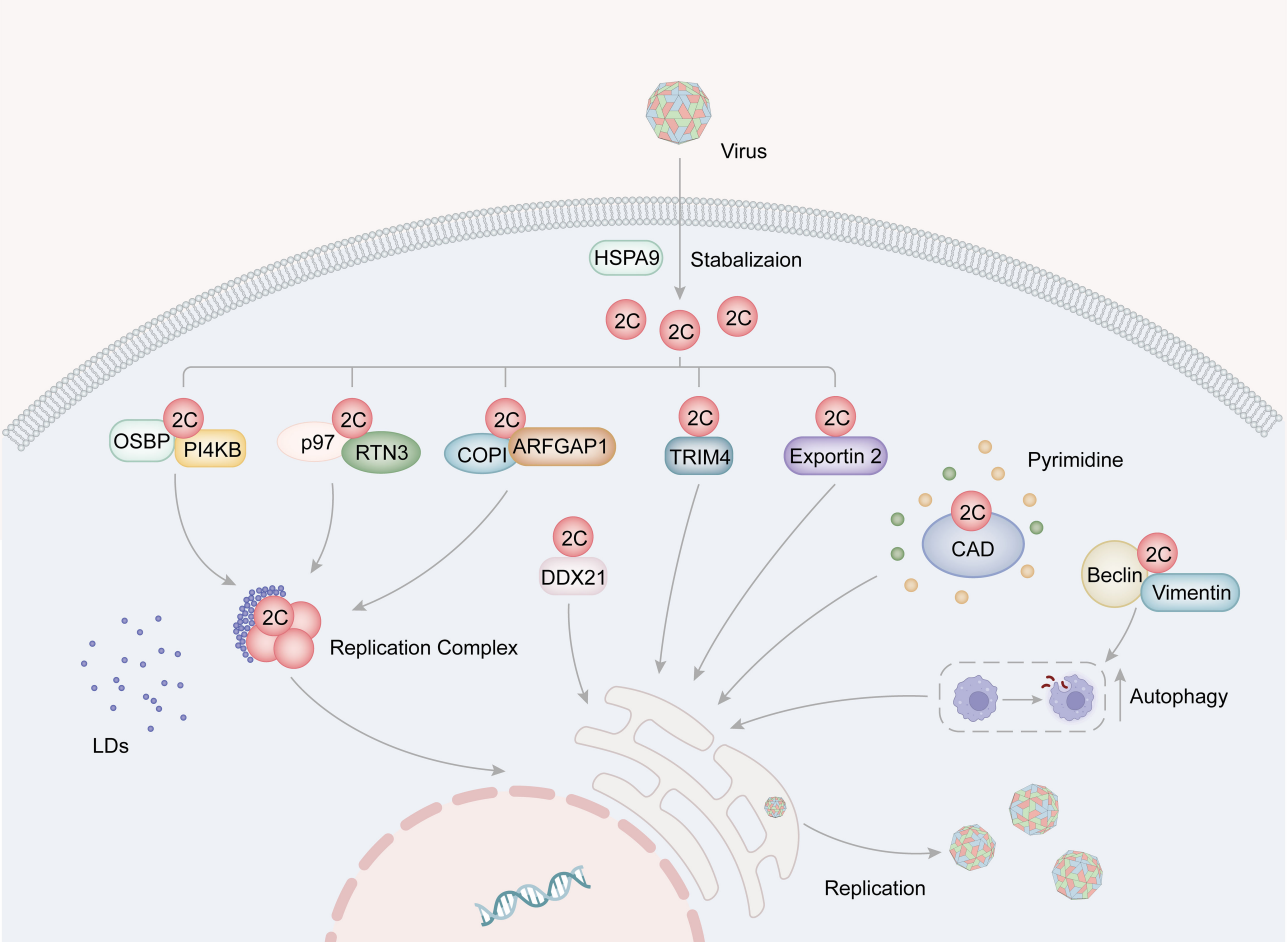
The role for picornavirus 2C protein in viral replication. The HSP family of proteins plays a role in stabilizing the structure of 2C. Specifically, EV-A71 2C binds to VCP/p97 and RTN3, thereby facilitating the formation of the replication complex. Additionally, the interaction of 2C with the OSBP-PI4KB complex and the COPI complex promotes the accumulation of lipid droplets around the replication complex, consequently supporting viral replication. Moreover, FMDV 2C interacts with CAD proteins to facilitate pyrimidine synthesis, and also engages with Beclin1 and vimentin to induce cellular autophagy, thereby providing essential resources for viral replication. Furthermore, 2C binds to host factors such as TRIM4, exportin2, and DDX21, all of which contribute to the enhancement of viral replication.

The positive-stranded RNA genome of Picornaviruses, which is released from the viral capsid, acts as a template for both replication and translation processes. Viral translation occurs through an IRES-dependent mechanism and leads to the cleavage of cytokines and rearrangement of cellular membranes. The replication complex (RC) is a crucial component involved in viral replication and consists of 2B, 2C (or its precursors 2BC), 3A (or its precursors 3AB), 3D^pol^, and host factors. These components have been found to be associated with virus-induced membrane vesicle (Ilnytska et al., 2013; van der Schaar et al., 2016). 2C is a crucial constituent of RC and serves an important role in viral replication. The interaction between host factors and 2C proteins is essential during replication as it provides materials, RC as a stable replication platform, and other foundations for viral replication. Some host factors have been identified to promote viral replication through different mechanisms by interacting with 2C proteins (**Figure 3**).

Picornaviruses typically induce infected cells to form new cytoplasmic vesicular compartments as replication sites instead of using the original organelle membrane (Bienz et al., 1994; van der Schaar et al., 2016). The RC of enteroviruses is formed by new membrane contact sites that attract host LDs to the RC. Viral proteins connect the RC to the lipid droplets and facilitate the lipid breakdown in the host cell, providing fatty acids as raw material for RC formation.

The phosphatidylinositol-4 kinase IIIb (PI4KB) and oxysterol-binding protein (OSBP) family proteins have been found to be vital for the formation of the RC of Picornaviruses (Arita, 2014, 2016; Banerjee et al., 2018). For instances, the Aichi virus (AiV) 2BC and 2C proteins bind to acyl-coenzyme A binding domain containing 3 (ACBD3) and PI4KB, consequently formed virus protein/ACBD3/PI4KB complex, which is located at AiV RNA replication sites and promotes PI4KB-dependent phosphatidylinositol 4-phosphate (PI4P) production (Sasaki et al., 2012). Interactions between AiV 2BC, 2C proteins, and components of the PI4KB/OSBP pathway, including OSBP, VAP-A/B, and SAC1, facilitate the recruitment of these proteins into the AiV RO and are essential for viral replication (Ishikawa-Sasaki et al., 2018). However, PV can evolve to become less dependent on this host pathway through specific mutations, such as the 2C-M187V mutation, which promotes viral replication, growth, and spread (Arita, 2021).

ER, Golgi apparatus, mitochondria, and endolysosomal compartments in host cell can serve as sources of membrane components for viral replication zone vesicles (Salonen et al., 2005). The Reticulon (RTN) family contributes to the stabilization of the ER network, apoptosis promotion, protein secretion, and translocation of ER components (Oertle and Schwab, 2003; Voeltz et al., 2006; Yan et al., 2006). Among the four members of the RTN family, Reticulon 3 (RTN3) interacts with the N-terminal regions of 2C in picornaviruses such as EV-A71, PV and CV-A16 (Tang et al., 2007). The amino acid residue I25 of EV-A71 2C plays a critical role in its interaction with RTN3’s reticular homology domain (RHD). Furthermore, the PV 2C I25K mutation modulates viral protein processing and RNA replication (Paul et al., 1994). EV-A71 2C also interacts with RHDs in C-terminal of other members in the RTN family (Tang et al., 2007).

Certain host factors promote the formation of RCs by stabilizing relevant proteins, such as HSPA9 and valosin-containing protein (VCP/p97) (Wang et al., 2017; Su et al., 2020). Heat shock proteins 70 (HSP70s), including HSPA9, play diverse roles in various stages of the viral life cycle. HSPA9 assists in folding and stabilizing 2C proteins, thereby contributing to the formation of RCs (Su et al., 2020). In mammalian cells, the ER protein quality control (ERQC) system plays a crucial role in guaranteeing the proper folding and assembly of proteins, ensuring their successful delivery to their designated locations during the process of maturation (Wiseman et al., 2022). The ubiquitin-proteasome-mediated ER-associated degradation (ERAD) pathway is one of three pathways within the ERQC system (Christianson et al., 2023). VCP, a component of the ERAD pathway, co-localizes with EV-A71 2C and RTN3, suggesting its involvement in viral replication (Wang et al., 2017). Additionally, VCP exhibits co-localization with PV proteins 2BC/2C and 3AB/3B, interacts with 2BC and 3AB, and plays a crucial role in facilitating PV replication and the cellular protein secretion pathway throughout the course of PV infection (Arita et al., 2012).

The coat protein complex I (COPI) vesicles facilitate the retrograde transport of cargoes, including escaped ER-resident proteins, from the Golgi to the ER and maintain lipid homeostasis (Hsu et al., 2009). COPI activity is required for picornaviruses (Gazina et al., 2002; Belov et al., 2007; Lanke et al., 2009; Wang et al., 2012b; Moghimi et al., 2020). ARF1 is the small GTPase in COPI vesicular transport. ARFGAP1 functions as a GTPase-activating protein for ARF1 and has been identified as a binding partner of 2C. It has also been shown to be a host factor for EV-A71 (Li et al., 2019b). The interaction between the 2C and the key components of COPI machinery suggests that COPI may act on the viral RC through 2C protein (**Figure 3**).GBF1 exhibits guanine nucleotide exchange factor (GEF) activity toward ARF1 and is redistributed to enterovirus replication organelles through its interaction with the 3A protein. Research indicates that PV progressively develops resistance to increasing concentrations of brefeldin A (BFA), an inhibitor of GBF1 (Viktorova et al., 2023). The accumulation of multiple mutations in 2C leads to the development of high levels of resistance, even in the presence of BFA. This resistance was also observed in PV 3A mutants and GBF1-deficient cells, suggesting that aberrant recruitment of GBF1 to replicating organelles occurs in the presence of 3A mutation. Notably, activated ARF1 accumulates on the replication organelles of PV 2C mutants, even in the presence of BFA. Additionally, the pan-ARFGEF inhibitor LM11 suppressed replication, and knockdown of ARF1 affected cellular resistance to BFA (Viktorova et al., 2023). The study highlights that the mutations significantly enhance the interaction between 2C and the activated form of ARF1. Interestingly, HRV 2C demonstrates a particularly strong interaction with activated ARF1, resulting in less sensitivity to BFA compared to PV. This suggests that 2C may act as an effector protein for ARF1, and although GBF1 plays a significant role, the activation of ARF1 may not be essential for enterovirus replication.

Carbamoyl-phosphate synthetase 2, aspartate transcarbamylase, and dihydroorotase (CAD) is transcription factor that operates downstream of the AKTmTOR signaling pathway. It serves as crucial proteins in the process of pyrimidine *de novo* synthesis (Moreno-Morcillo et al., 2017). When CAD is inhibited using short hairpin RNA (shRNA), it leads to the disruption of FMDV RNA and protein levels (Yang et al., 2023). Within FMDV-infected cells, the ACTase domain of CAD interacts with FMDV 2C and colocalizes with 2C in the replication complex (RC). This suggests that Picornavirus potentially recruits CAD through the 2C protein to ensure an adequate supply of pyrimidines during the replication process (Yang et al., 2023).

Autophagy facilitates the degradation of protein aggregates and impaired organelles by sequestering them within double-membrane vesicles that eventually fuse with autolysosomes (Tanida, 2011). Many Picornaviruses, including EV-A71, CV, coronaviruses, dengue viruses, influenza A viruses, and PV, exploit cellular autophagy to promote viral replication (Choi et al., 2018). Beclin 1 and vimentin participate in cellular autophagy, which is crucial for viral replication. Interactions between 2C and beclin 1 and vimentin allow FMDV to take advantage of autophagy’s positive regulation (Gladue et al., 2013). Additionally, vimentin protein appears around 2C after FMDV infection, aiding in viral replication. Interestingly, although the overexpression of vimentin does not impact viral replication, the overexpression of truncated dominant-negative waveform proteins notably reduces viral loading (Gladue et al., 2013).

The Asp-Glu-Ala-Asp (DEAD) motif possesses catalytic properties essential for ATP hydrolysis, and DEAD-box helicases (DDXs) that contain this motif play various roles in translation, transcription, RNA degradation, and apoptosis (Meier-Stephenson et al., 2018; Taschuk and Cherry, 2020). Overexpression of FMDV 2C leads to the degradation of DDX21 through lysosomal and cystatin pathway-dependent mechanisms, negatively regulating FMDV IRES-dependent translation and replication (Abdullah et al., 2021). Additionally, TRIM4 and exportin2 have been confirmed as binding partners of 2C and demonstrated to be new host factors for EV-A71 (Li et al., 2019b).

### 4.3 The role for 2C in modulating innate immunity

**Figure 4:**
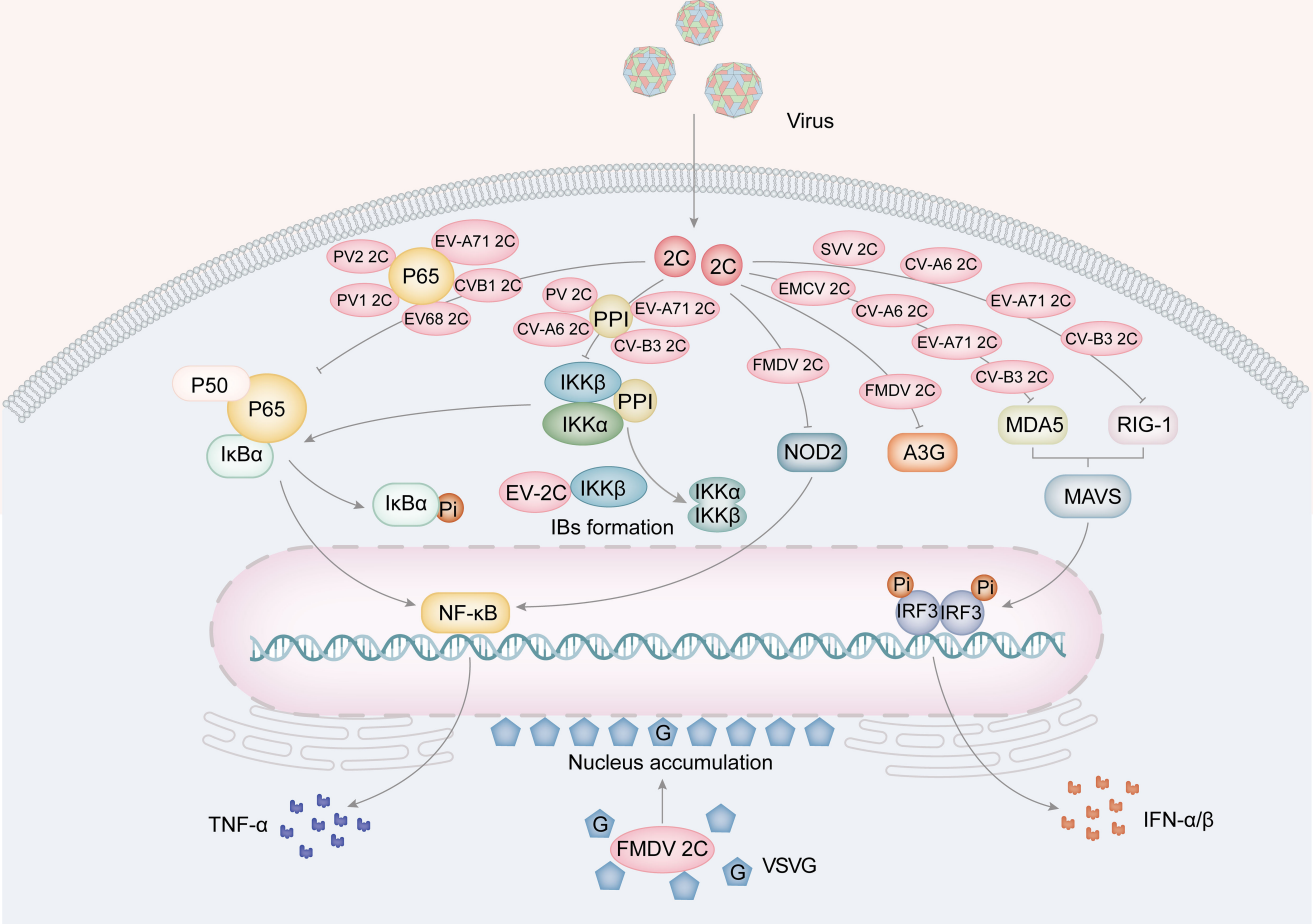
The role for picornavirus 2C protein in innate immunity. The 2C protein of EV68, EV-A71, PV1, PV2 and CV-B3 is implicated in the suppression of TNF-α by hindering the formation of the NF-κB-p65/p50 complex and the IKK complex (PV, EV-A71, CV-A6 and CV-B3). Furthermore, FMDV 2C also suppresses A3G and the expression of NOD-2, a protein involved in the NF-κB pathway. Furthermore, 2C proteins from EMCV, CV-A6, EV-A71, and CV-B3 have been found to inhibit MDA5, while 2C proteins from SVV, CV-A6, EV-A71 and CV-B3 inhibit RIG-I. These inhibitory actions result in a suppression of IFNα/β production. Moreover, FMDV 2C protein facilitates the aggregation of the VSVG protein in the perinuclear area.

Besides promoting viral replication, 2C proteins contribute to innate immunity (**Figure 4**). The host innate immune system is the initial defense system against viral infection. It prevents viruses from invading or multiplying before the adaptive immune system can produce a more specific protective effect (Koyama et al., 2008). Studies have shown that following infection with FMDV, expression of 2BC or co-expression of 2B and 2C will lead to a decrease in major histocompatibility complex I (MHC I), detectable as early as half an hour after infection and halved within 6 hours (Sanz-Parra et al., 1998; Moffat et al., 2005, Moffat et al., 2007). Furthermore, the overexpression of FDMV 2C causes an accumulation of the vesicular stomatitis virus G protein (VSVG) at perinuclear sites and hinders its transport to vesicles located on the cell surface (Moffat et al., 2005, Moffat et al., 2007).

Pattern Recognition Receptors (PRRs) are responsible for detecting specific components of viruses and play a role in the innate immune response through three pathways (Jin et al., 2018). These pathways involve the detection of pathogen-associated molecular patterns (PAMPs), counteracting the metabolic consequences of infection and inflammation through danger-associated molecular patterns (DAMPs), and recognizing “missing self” molecules from healthy cells or microbes (Jin et al., 2018). The three main types of PRRs are RIG-I-like receptors (RLRs), Toll-like receptors (TLRs), and NOD-like receptors (NLRs). Additionally, certain secreted functional proteins promote the production of type I interferons (IFNs) and proinflammatory factors, which are important in viral infections (Akira et al., 2006).

The RLRs family consists of retinoic acid-inducible gene I (RIG-I), melanoma differentiation-associated protein 5 (MDA5), and laboratory of genetics and physiology 2 (LGP2) (Bruns and Horvath, 2014). RIG-I and MDA5 contain caspase activation and recruitment domains (CARDs), an ATPase/helicase domain, and a regulatory/repression domain (Zheng et al., 2023). These proteins activate the interferon signaling pathway by recognizing viral infections and interacting with the mitochondrial antiviral signaling protein (MAVS). This interaction leads to the activation of IFN regulatory factor 3 (IRF3) and nuclear factor-κB (NF-κB), which stimulate the expression of type I IFNs, interferon-stimulated genes (ISGs), and inflammatory cytokines (Sharma et al., 2021). The 2C protein can weaken the host’s immune system by targeting RIG-I. Studies have shown that the 2C and 3C proteins of Seneca Valley virus (SVV) degrade RIG-I via the caspase signaling pathway, reducing the production of IFN-β (Wen et al., 2019; Liu et al., 2019b). Similarly, the EMCV 2C protein interacts with MDA5 to inhibit the expression of IFN-β. Mutations in the EMCV 2C protein can affect its ability to inhibit MDA5/RIG-I, leading to reduced viral replication and release (Li et al., 2019a).

In HEK 293 T cells, the 2C protein in CV-A6, EV-A71, and CV-B3 hinders the production of IFN-β by interfering with MDA 5 and RIG-I (**Figure 4**) (Wang et al., 2023). This interference occurs through the interaction of 2C with MDA 5 and RIG-I, resulting in their degradation through the lysosomal pathway. In the case of CV-A6, 2C does not recognize MDA5 and RIG-I, rendering the previous conclusion invalid (Wang et al., 2023). Significantly, mutations of F28A, V75A, and I96V in CV-A6 2C greatly reduce its capacity to inhibit MDA5/RIG-I. While V75A and I96V disrupt the interaction of CV-A6 2C with MDA5/RIG-I, F28A retained its competency in binding to MDA 5/RIG-I, suggesting that merely the interaction is insufficient for the 2C-induced decrease. Additionally, the decreased efficacy of IFN-β inhibition by CV-A6 2C F28A is linked to the attenuation of viral replication and release (Wang et al., 2023).

NF-κB is a crucial nuclear transcription factor that regulates the expression of proinflammatory cytokines, such as TNF-α and IL-6 (Karin, 2009). To avoid the host immune response, viruses manipulate the NF-κB pathway to enhance viral replication and cell survival. The 2C protein is crucial in regulating the NF-κB pathway by engaging with the IκB kinase (IKK) complex of the NF-κB pathway, which involves phosphorylating IKKα and IKKβ. This leads to the phosphorylation of the IκB protein, a suppressor of NF-κB transcription factors. EV-71 2C directly interacts with the kinase domain (KD) of IKKβ via the N-terminal 1-125AA and inhibits IKKβ phosphorylation, thereby blocking TNF-α-mediated NF-κB activation (**Figure 4**) (Zheng et al., 2011). Among the NF-κB family, NF-κB p65/p50 heterodimers are the most abundant signaling complexes (Rahman and McFadden, 2011). Their translocation to the nucleus stimulates the release of cytokines and chemokines, which are vital for the host’s innate immune response against viral infections (Fagerlund et al., 2005). The 105-125 and 126-203 AA of the EV-A71 2C interact with the IPT domain of p65, leading to the inhibition of p65/p50 heterodimer formation (**Figure 4**) (Du et al., 2015). Further research has revealed that EV-A71 2C can sequester IKKα and IKKβ into inclusion bodies (IBs) through direct interaction with IKKβ. At the same time, EV-A71 2C can inhibit the phosphorylation activity of IKKα through its interaction with IKKβ (Ji et al., 2021). EV-A71 2C, CV-A16 2C, CV-B3 2C, and PV 2C form a complex by recruiting PP1 and IKK, inhibiting the NF-κB signaling pathway (**Figure 4**) (Zheng et al., 2011; Li et al., 2016).

In addition to RLRs, NLRs have been identified as essential in antiviral immune response (Lupfer and Kanneganti, 2013). Nucleotide-binding oligomerization domain 2 (NOD2) was found to be a cytoplasmic viral PRR, and FMDV activates the NOD2-mediated IFN-β and NF-κB signaling pathways (Liu et al., 2019a, 2021). FMDV 2C is responsible for reducing the levels of NOD2 protein, independent of host eukaryotic translation initiation factor 4 gamma (eIF4G) cleavage, cellular apoptosis induction, or involvement of proteasome, lysosome, and caspase pathways (**Figure 4**). The interaction between FMDV 2C and NOD2 is critically dependent on the C-terminal amino acid pair at positions 116-260. Furthermore, 2C proteins can impede the host cell’s innate immune response through alternative pathways. For instance, EV-A71 2C can trigger A3G degradation via the autophagy-lysosome pathway (**Figure 4**) (Li et al., 2018b).

## 5 Inhibitors of 2C

2C protein inhibitors can modulate cell signaling pathways by suppressing the activity of 2C proteins, thereby inhibiting infections. The researchers’ objective is to create highly selective and powerful drugs that can effectively inhibit the activity of 2C proteins, thus intervening in the progression of diseases.

### 5.1 GuHCl

Guanidine hydrochloride (GuHCl) has been found to inhibit the replication of picornaviruses and other viruses, including EV, PV, CV, FMDV, Ebola virus (EBOV), echoviruses, and noroviruses by blocking viral RNA synthesis (Tershak, 1982; De Palma et al., 2008b; Xia et al., 2015; Li et al., 2018a; Shu et al., 2019). Researchers found that GuHCl targets PV 2C, blocking RNA (negative strand only) synthesis and RNA strand elongation (Barton and Flanegan, 1997). Resistance and reliance of PV and FMDV to GuHCl can be attributed to mutations 179 and 187 of 2C (Saunders et al., 1985; Pincus et al., 1986; Baltera and Tershak, 1989; Barton and Flanegan, 1997). Additionally, Pfister et al. discovered that guanidine hydrochloride inhibits viral RNA replication by interfering with the ATPase activity of PV 2C (Pfister and Wimmer, 1999). Subsequent studies indicated that millimolar concentrations of GuHCl can inhibit ATP hydrolyzing activity of PV 2C (Pfister et al., 2000). GuHCl has also been reported to inhibit the NTPase and deconjugase activities of the EV-A71 2C (Xia et al., 2015). Interestingly, GuHCl has been found to inhibit the RNA remodeling activities of other viral proteins, including the NS3 protein of human norovirus and the VP35 protein of EBOV (Li et al., 2018a; Shu et al., 2019). Norovirus NS3 possesses ATP-dependent RNA helicase activity and ATP-independent RNA-chaperoning activity (Li et al., 2018a), while EBOV VP35 exhibits NTP-dependent RNA helicase-like activity (Shu et al., 2019). GuHCl binds to the surface of viral RNA-remodeling proteins, altering their conformation, electrostatic state, and interactions, thereby inhibiting their RNA-remodeling activity.

### 5.2 Fluoxetine

Targeting the 2C protein, fluoxetine is a selective 5-hydroxytryptamine reuptake inhibitor. It is a non-broad-spectrum anti-enterovirus inhibitor. Initially, fluoxetine was found to selectively inhibit replication of EV-B and EV-D only, and was ineffective against EV-A, EV-C, RV-A or RV-B (Ulferts et al., 2013). Therefore, there is a need to develop safer and more effective small-molecule drugs (Hu et al., 2020). Both (S)-fluoxetine and racemic fluoxetine mixtures are ineffective against EV-A71 but can inhibit CVB3 and EV-D68, and the degree of inhibition is more significant with (S)-fluoxetine (Hurdiss et al., 2022). However, neither the racemic mixture nor the individual enantiomers of fluoxetine were active against EV-A71 (Bauer et al., 2019). Recent studies have shown that fluoxetine 12a, 19b, and 19d inhibit EV-A71, CV-B3, PV-1, CV-A24, EV-D68, HRV-A2, and HRV-B14.

In 2019, Bauer et al. reported that (S)-fluoxetine has a higher CV-B3 2C binding affinity than (R)-fluoxetine and was able to inhibit enterovirus replication (Bauer et al., 2019). (S)-fluoxetine stabilizes the 2C protein hexamer by binding to the CV-B3 2C 117-329 site (Hurdiss et al., 2022). The resistance mutations in the 2C protein are located in the sites bound to (S)-fluoxetine (C179F, C179Y, and F190L) and in the 224-AGSINA-229 loop. The 224-AGSINA-229 loop, which includes A224V, I227V, and A229V, resides downstream of the C motif of the ATPase and has no direct interaction with (S)-fluoxetine (Hurdiss et al., 2022). The 224-AGSINA-229 ring is conserved in nonresistant EV-B, CV-B3, and EV-D68 but not in other species of enteroviruses, implying that some of the mutations in 2C may contribute to fluoxetine resistance. If substitutions occur at the A224, I227, and A229 sites, a person will develop resistance to fluoxetine. Also, resistance to drugs such as TBZE-029, HBB, MRL-1237, and GuHCl is also affected. Subsequent studies on resistant mutants have shown that inhibitors act by binding to the α2 helix of the 2C (Bauer et al., 2020). And (S)-fluoxetine locks the 2C hexamer state to rationalize its inhibition mode (Hurdiss et al., 2022).

### 5.3 Dibucaine

Dibucaine is a local anesthetic that inhibits Na^+^ channels and has analgesic effects. Additionally, it acts as an antiviral inhibitor targeting the 2C protein. Initially, Musharrafieh et al. discovered enhanced efficacy of quinoline derivatives chemically modified from dibucaine against EV-D68. And further modification of the quinoline analogs significantly improved their antiviral activity and selectivity index against virus. Importantly, the compound 12a did not inhibit Na+ channels, thus alleviating concerns about its analgesic effects (Musharrafieh et al., 2019).

Several researchers conducted structure-activity relationship (SAR) study, searching for dibucaine analogs with higher antiviral activity and selectivity (Tang et al., 2020). They found that oral administration of a low-toxicity derivative of dibucaine, 6i, protected EV-A71-infected mice. Furthermore, when combined with the IRES inhibitor emetine, compound 6i exhibited a synergistic effect in completely preventing and treating the symptoms of EV-A71-infected mice.

There are other 2C protein inhibitors, including 2CL, B-2CL, TBZE-029, mefendil, and MRL-1237 (De Palma et al., 2008a; Smee et al., 2016). 2CL and B-2CL are small molecule compounds designed on the basis of the 2C structure of EV-A and EV-B. They can target 2C proteins and effectively inhibit the oligomerization of 2C proteins. Experimental evidence has been shown that 2CL protects EV-A71-infected mice (Fang et al., 2021). The growth of EV-B and EV-D is suppressed by TBZE-029, whereas EV-A and EV-C are unaffected by its inhibitory effects (De Palma et al., 2008a). Methylfuryl alcohol and N6-phenyladenosine have shown inhibition against EV-A71 with a resistance mutation in the 2C protein, although no direct binding assay was available (Smee et al., 2016).

## 6 Conclusion and prospect

Picornaviruses, small, non-enveloped, positive-stranded RNA viruses, are accountable for a wide range of illnesses in humans and animals. Those results in a substantial healthcare burden, with hundreds of millions of infections reported each year. The 2C proteins, among the nonstructural proteins in picornaviruses, are highly conserved and represent a focal point of interest for researchers. This review provides a thorough examination of the relationship between the structure and function of 2C proteins in picornaviruses, delving into their involvement in virus morphology, replication, interactions with host proteins related to innate immunity, and inhibitors targeting 2C proteins. The multifaceted 2C proteins comprise diverse structural domains that perform indispensable functions throughout the viral life cycle, influencing viral replication, packaging, and infection. The review consolidates the current comprehension of the amphipathic domain, ATPase domain, and zinc finger domain of enterovirus 2C proteins and their respective structure-function relationships, with future studies aiming to explore the intricate mechanisms of different structural domains involved in the viral infection process, thereby contributing to a deeper understanding of picornaviruses.

The interactions between picornavirus 2C proteins and various host factors such as RTN3, COPI, PI4KB, NF-κB, MDA5, RIG-I, NOD2, and IFN signaling pathways have been elucidated, emphasizing the potential for identifying new targets for antiviral therapy development. Future research should aim to uncover additional host factors interacting with 2C proteins and delve into the pathways through which these factors act, particularly focusing on the NF-κB pathway and three major PRRs, to better understand the mechanism of action of 2C proteins.

Furthermore, this review has highlighted broad-spectrum antiviral drugs targeting enterovirus infections by interacting with 2C proteins. However, there is a need for deeper insights into the precise mechanism of action of these drugs and the development of new 2C protein inhibitors, considering multi-target effects and optimizing drug properties. These efforts will open up new possibilities for both research and clinical applications of 2C protein inhibitors, ultimately enhancing patient treatment options in the future.

## Author contributions

CY: Writing – original draft. HZ: Writing – original draft. XX: Writing – review & editing. ZP: Writing – review & editing. DL: Writing – review & editing. LZ: Conceptualization, Writing – review & editing.
